# Sex Differences in QT Interval and Beat-to-Beat Temporal QT Variability at Rest and After Submaximal Exercise in Young Adults With and Without Obesity: A Cross-Sectional Study

**DOI:** 10.7759/cureus.118007

**Published:** 2026-10-16

**Authors:** Sujoy Mukherjee, Goutam Banerjee, Yogesh Kumar

**Affiliations:** 1 Physiology, West Bengal Medical Education Services, Kolkata, IND; 2 Physiology, All India Institute of Medical Sciences Patna, Patna, IND; 3 Physiology, Sarat Chandra Chattopadhyay (SCC) Government Medical College and Hospital, Uluberia, IND

**Keywords:** body mass index, exercise, obesity, qt dispersion, qt interval, sex differences, ventricular repolarization, young adults

## Abstract

Background

Obesity increases arrhythmia risk by altering QT dynamics. However, sex-specific differences during rest and exercise remain insufficiently characterized in young adults, representing a critical gap in cardiovascular risk research.

Objectives

This study aimed to (1) evaluate sex differences and the potential interaction between sex and obesity in heart rate-corrected QT interval (QTc) and single-lead temporal beat-to-beat QT variability (QTvar) at rest and following submaximal exercise and (2) explore subgroup-specific relationships between body mass index (BMI) and repolarization parameters.

Methods

A cross-sectional observational study was conducted on 200 healthy young adults (aged 18-25 years) categorized into obese and non-obese groups based on World Health Organization Asia-Pacific BMI criteria. Participants underwent resting and post-exercise (50% VO2max) continuous Lead II electrocardiogram (ECG) recordings following a standardized submaximal treadmill protocol. QTc (using Bazett's formula) and temporal beat-to-beat QTvar (QTvar = QTmax - QTmin across 10 consecutive complexes) were quantified from Lead II. Statistical comparisons with Bonferroni adjustments and correlation analyses between BMI and QT parameters were performed.

Results

Obese females demonstrated significantly higher resting QTvar values than obese males both at rest (227.2 ± 68.5 vs. 174.6 ± 57.2 ms, p = 0.0002, Cohen's d = 0.83) and post-exercise (214.0 ± 25.4 vs. 184.6 ± 81.6 ms, p = 0.045, Cohen's d = 0.48). QTc intervals were numerically longer in females across groups, though differences were not statistically significant (p > 0.05). Following Bonferroni correction for multiple comparisons across correlation analyses (α = 0.0031), a significant positive correlation between BMI and post-exercise QTc was observed in obese females (r = 0.572, p = 0.0004), whereas correlations between BMI and resting QTc in females (r = 0.402, p = 0.018) and recovery QTc in obese males (r = 0.370, p = 0.005) represented exploratory associations that did not meet strict multiplicity adjustment.

Conclusions

Obese young females exhibit greater temporal beat-to-beat QTvar than obese males, suggesting sex-specific differences in dynamic ventricular repolarization stability under conditions of elevated adiposity. The emergence of a robust positive correlation between BMI and recovery QTc in obese females highlights the potential for differential autonomic and electrophysiological responses following physical exertion, providing hypothesis-generating evidence that warrants prospective longitudinal study.

## Introduction

Obesity has reached epidemic proportions globally, affecting increasingly younger populations and contributing significantly to cardiovascular morbidity and mortality [1]. The relationship between obesity and sudden cardiac death (SCD) is well-established, with multiple epidemiological studies demonstrating increased risk among obese individuals [2,3]. This heightened risk may be partially mediated through obesity-related alterations in cardiac electrophysiology, particularly affecting ventricular repolarization [4].

The electrocardiogram (ECG) provides a non-invasive method for assessing cardiac electrical activity and identifying individuals at risk for arrhythmias [5]. Among ECG parameters, the QT interval - representing the duration of ventricular depolarization and repolarization - serves as a critical marker of cardiac electrical stability [6]. Prolongation of the QT interval, particularly when corrected for heart rate (heart rate-corrected QT interval (QTc)), is associated with increased risk of ventricular arrhythmias and SCD [7,8]. In clinical electrocardiography, regional variations in repolarization have historically been evaluated using spatial QT dispersion across multiple leads [9,10]; however, dynamic temporal beat-to-beat variability in the QT interval within a single continuous lead (QT variability (QTvar)) provides complementary information regarding cycle-to-cycle repolarization stability and autonomic responsiveness [10].

Physiological sex differences in cardiac repolarization are well-documented, with post-pubertal females typically exhibiting longer QT intervals than males due to hormonal influences on cardiac ion channels [11,12]. However, the interaction between sex, obesity, and exercise-induced changes in QT dynamics remains insufficiently characterized, particularly in young, apparently healthy individuals. Previous studies have demonstrated obesity-related QT prolongation [13,14], but most investigations have not systematically examined sex-specific differences or the acute effects of exercise in this population.

To address these knowledge gaps, the present study was structured around two primary aims: (1) to evaluate sex differences and the potential interaction between sex and obesity in QTc and beat-to-beat temporal QTvar at rest and following submaximal exercise and (2) to examine exploratory relationships between BMI and repolarization parameters within sex-obesity subgroups. We hypothesized that females would display longer QTc intervals and greater beat-to-beat QTvar compared to males, that obesity would amplify these sex differences, and that BMI would correlate differentially with repolarization indices between sexes.

## Materials and methods

Study setting and ethical approval

This cross-sectional observational study was conducted in the research laboratory of the Department of Physiology at R.G. Kar Medical College and Hospital, Kolkata, India, following formal review and approval by the Institutional Ethics Committee. The study was conducted in accordance with the 2013 revision of the Declaration of Helsinki [15]. Written informed consent was obtained from all participants prior to enrollment.

Sample size estimation

The sample size was calculated based on expected differences in temporal QTvar between obese and non-obese individuals derived from preliminary data. Assuming a mean difference of 40 milliseconds (ms) with a pooled standard deviation (SD) of 70 ms, an alpha level of 0.05, and a power of 80%, the minimum required sample size was calculated as 39 participants per group using the following formula:

 

$$n = 2\left[\frac{(Z_\alpha + Z_\beta)\sigma}{\Delta}\right]^2$$

To account for potential data quality issues and to ensure adequate representation across sex and body mass index (BMI) categories, a total of 200 participants were enrolled.

Study population and recruitment

Participants were recruited through institutional advertisements and voluntary enrollment among medical students aged 18 to 25 years. According to the World Health Organization criteria for Asian populations [16], BMI categories are defined as underweight (<18.5 kg/m²), normal weight (18.5-22.9 kg/m²), overweight (23.0-24.9 kg/m²), and obese (≥25.0 kg/m²). In this study, non-obese status was defined as a BMI < 23.0 kg/m² (encompassing underweight and normal weight individuals), while obesity was defined as a BMI ≥ 25.0 kg/m². Individuals falling within the intermediate overweight range (23.0-24.9 kg/m²) were excluded during initial screening to establish a clear physiological separation between the non-obese and obese cohorts. The final cohort comprised 200 participants categorized into four groups: obese males (n = 54), obese females (n = 34), non-obese males (n = 46), and non-obese females (n = 66). The lower enrollment in the obese female cohort (n = 34) reflected the lower baseline prevalence of obesity among female medical students in this demographic and scheduling constraints associated with menstrual cycle timing. All participants were normotensive, non-smokers, and non-alcoholic, and none were engaged in structured athletic training or competitive sports. Female participants had regular menstrual cycles (26-32 days) and were evaluated strictly during the early-to-mid follicular phase (days 5 to 12) to minimize cyclic estrogen and progesterone fluctuations. Exclusion criteria included known cardiovascular, metabolic, or endocrine diseases (e.g., hypertension, diabetes mellitus, dyslipidemia), history of syncope or arrhythmias, current use of medications known to affect cardiac conduction or repolarization, acute illness within the preceding three weeks, pregnancy, or lactation.

Pre-test standardization and hemodynamic assessment

Participants were instructed to avoid caffeine and alcohol for 24 hours, abstain from strenuous physical exertion for 48 hours, and arrive in a fasting state (minimum four hours). Height was measured to the nearest 0.1 cm using a stadiometer, and body weight was recorded to the nearest 0.1 kg on a calibrated digital scale. BMI was computed as weight in kilograms divided by height in meters squared. Hemodynamic assessments were performed following 30 minutes of supine rest in a quiet, temperature-controlled room (22-24°C). Blood pressure was measured three times at five-minute intervals using a calibrated aneroid sphygmomanometer, and the mean of the final two measurements was used for analysis.

Electrocardiographic recording and interval measurement

A standard 12-lead electrode configuration was initially applied to verify normal sinus rhythm and exclude baseline conduction abnormalities or ischemic ST-T changes. Quantitative rhythm data were continuously recorded from Lead II using a calibrated Physiograph Polyrite-D system equipped with bio-amplifiers (four-channel, Model No.: RMS/EN-ISO 13485:2003, Recorders and Medicare Systems, Chandigarh, India) at a paper sweep speed of 25 mm/s and voltage amplitude calibration of 10 mm/mV (0.1 mV/mm). Lead II was selected because of its prominent, upright T-wave morphology and well-defined baseline return, which optimize QT boundary identification [17]. To minimize observer bias, all digital tracings were de-identified, stripped of participant demographic data and condition labels (“rest” vs. “recovery”), and assigned randomized alphanumeric codes. Tracings were analyzed in non-consecutive, randomized batches by two independent physiologists blinded to participant characteristics and trial phase. QT intervals were quantified from 10 consecutive, artifact-free cardiac cycles using standardized digital tangent calipers. The QT interval was measured from the earliest onset of the QRS complex to the intersection of the maximum isoelectric baseline with the steepest downward slope of the T-wave. When U-waves were present, the nadir between the T-wave and U-wave was used as the terminal boundary. Discrepancies between observers exceeding 20 ms were adjudicated through consensus review with an independent senior physiologist.

Heart rate correction was performed using Bazett's classical formula [18]:

$$\mathrm{QTc} = \frac{\mathrm{QT}}{\sqrt{\mathrm{RR}}}$$

where RR represents the preceding R-R interval in seconds, while Bazett's square-root formulation is recognized to overcorrect at higher heart rates [19], it was retained to allow direct comparison with existing published young adult literature. Single-lead temporal beat-to-beat QTvar, capturing dynamic cycle-to-cycle variation across consecutive cardiac cycles, was calculated as QTvar = QTmax - QTmin, where QTmax and QTmin represent the longest and shortest QT intervals measured across the 10 consecutive complexes in Lead II.

Submaximal exercise protocol

Following resting evaluations, participants performed submaximal aerobic exercise on a motor-driven treadmill using a modified Bruce protocol (commencing at 2.7 km/hour at a 10% incline, with progressive incremental workload stages every three minutes). Workload was titrated until participants attained sex-specific target heart rates corresponding to approximately 50% of predicted maximal oxygen uptake (VO2max), calculated using the age-adjusted Åstrand-Ryhming nomogram (128 beats/minute for males and 138 beats/minute for females). Upon reaching the target heart rate, the treadmill workload was kept constant for five minutes to achieve steady-state aerobic exercise. Immediately upon exercise completion, participants were assisted to an adjacent couch and positioned in a quiet supine recovery state. Continuous monitoring was maintained across a 15-minute recovery period, and a standardized five-minute continuous Lead II ECG recording was acquired specifically during minutes 10 to 15 of recovery. This timeframe was selected to capture post-exercise electrophysiological recovery after the initial acute phase of autonomic deceleration and motion artifact had resolved. The measurement protocol used for resting tracings was applied identically to the recovery tracings.

Statistical analysis

Data distribution was assessed for normality using the Shapiro-Wilk test. Continuous variables are presented as mean ± SD. Differences between sexes within the obese and non-obese cohorts were evaluated using independent Student's t-tests. Effect sizes were calculated using Cohen's d, defined as the difference between group means divided by the pooled SD (s_pooled_):

$$s_{\mathrm{pooled}} = \sqrt{\frac{(n_1 - 1)s_1^2 + (n_2 - 1)s_2^2}{n_1 + n_2 - 2}}$$

For between-group comparisons across hemodynamic and electrocardiographic parameters (eight comparisons per cohort), a Bonferroni correction was applied (α = 0.05 / 8 = 0.00625). Relationships between BMI and QT parameters at rest and during recovery were assessed using Pearson correlation coefficients (r) with 95% confidence intervals. To account for multiple testing across correlation evaluations (16 separate tests), a Bonferroni-adjusted significance threshold of α = 0.05 / 16 = 0.003125 was established; correlations with nominal p < 0.05 that did not meet this threshold were interpreted cautiously as exploratory associations. Statistical analyses were executed using SPSS version 20.0 (IBM Corp., Armonk, NY) and GraphPad QuickCalcs (GraphPad Software, Boston, MA).

## Results

Participant characteristics

A total of 200 participants completed the study protocol without adverse events. The distribution across sex and obesity cohorts is illustrated in Figure 1.

**Figure 1:**
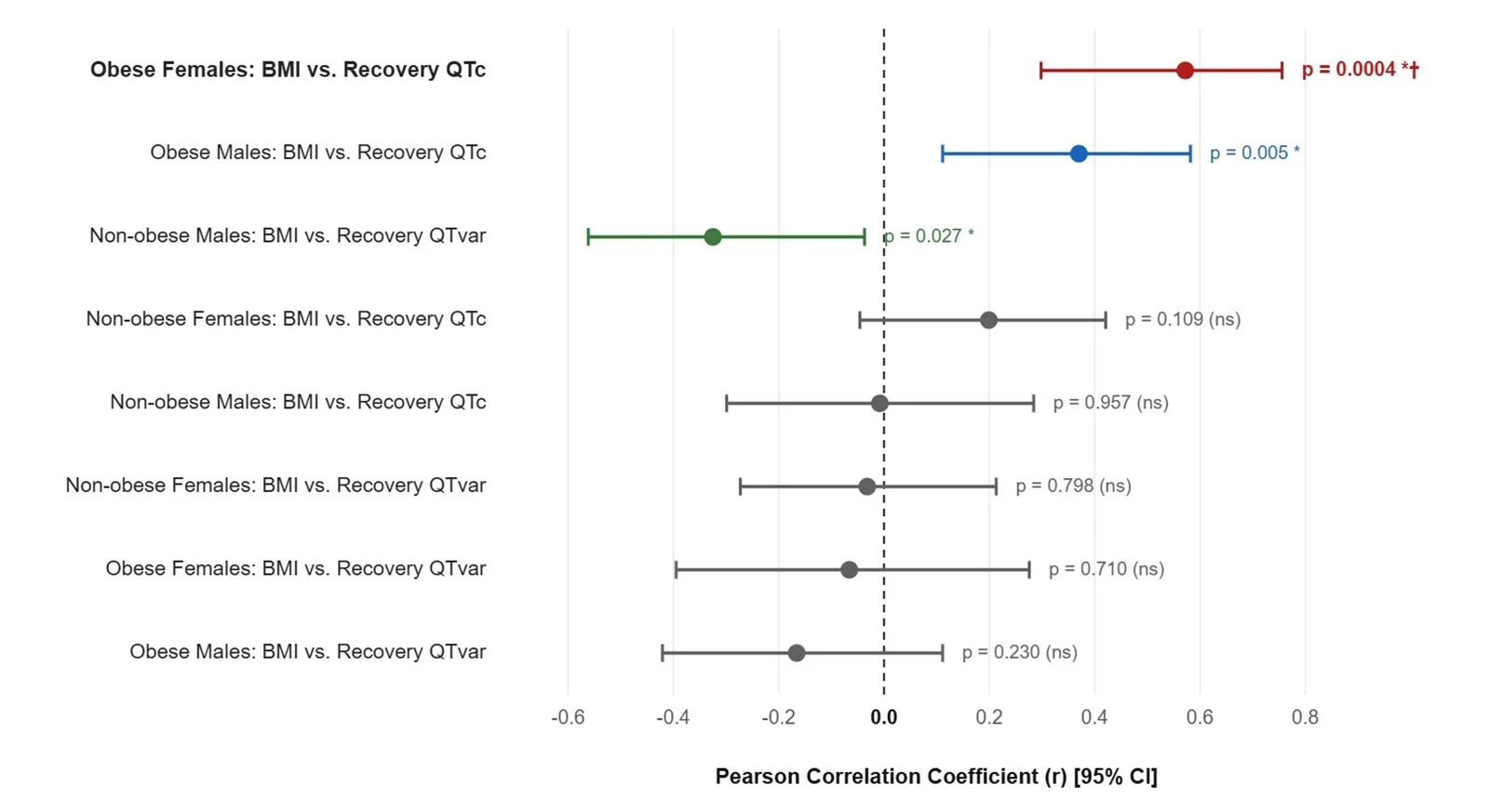
Subgroup Correlations Between BMI and Ventricular Repolarization During Recovery Pearson correlation coefficients (r) with 95% confidence intervals.

Baseline characteristics of the obese cohort are summarized in Table 1. Obese males and females were comparable in age (19.1 ± 1.5 vs. 18.7 ± 0.6 years, p = 0.127) and BMI (27.1 ± 3.4 vs. 26.5 ± 1.6 kg/m², p = 0.394). Obese males had significantly higher resting systolic blood pressure than obese females (125.6 ± 12.6 vs. 120.6 ± 6.8 mmHg, p = 0.037, Cohen's d = 0.48), whereas diastolic blood pressure did not differ significantly (78.8 ± 9.5 vs. 80.5 ± 1.9 mmHg, p = 0.321).

**Table 1:** Demographic, Anthropometric, and Electrocardiographic Characteristics of Obese Participants Data presented as mean ± SD. *p < 0.05. ^†^Remains statistically significant after Bonferroni correction (α = 0.00625 for eight comparisons). BMI: body mass index; BP: blood pressure; QTc: heart rate-corrected QT interval; QTvar: beat-to-beat temporal QT variability in Lead II

Variable	Obese Males (n = 54)	Obese Females (n = 34)	Mean Difference (95% CI)	p-value	Cohen's d
Age (years)	19.1 ± 1.5	18.7 ± 0.6	0.4 (-0.1 to 0.9)	0.127	0.33
BMI (kg/m²)	27.1 ± 3.4	26.5 ± 1.6	0.5 (-0.7 to 1.8)	0.394	0.19
Systolic BP (mmHg)	125.6 ± 12.6	120.6 ± 6.8	5.0 (0.3 to 9.7)	0.037*	0.48
Diastolic BP (mmHg)	78.8 ± 9.5	80.5 ± 1.9	-1.7 (-4.9 to 1.6)	0.321	0.24
Resting QTc (ms)	282.3 ± 75.8	299.5 ± 113.9	-17.3 (-58.9 to 24.3)	0.394	0.18
Resting QTvar (ms)	174.6 ± 57.2	227.2 ± 68.5	-52.6 (-81.6 to -23.6)	0.0002*^†^	0.83
Recovery QTc (ms)	280.2 ± 79.8	309.6 ± 108.9	-29.4 (-70.7 to 12.0)	0.149	0.30
Recovery QTvar (ms)	184.6 ± 81.6	214.0 ± 25.4	-29.4 (-56.2 to -2.6)	0.045*	0.48

Demographic, anthropometric, and hemodynamic characteristics of obese participants are presented in Table 1. Males and females were well-matched for age (19.1 ± 1.5 vs. 18.7 ± 0.6 years, p = 0.127) and BMI (27.1 ± 3.4 vs. 26.5 ± 1.6 kg/m², p = 0.394). Obese males demonstrated significantly higher systolic blood pressure compared to obese females (125.6 ± 12.6 vs. 120.6 ± 6.8 mmHg, p = 0.037, Cohen's d = 0.48), while diastolic blood pressure showed no significant sex difference (78.8 ± 9.5 vs. 80.5 ± 1.9 mmHg, p = 0.321).

Baseline characteristics of the non-obese cohort are shown in Table 2. Non-obese males and females showed a minor difference in age (19.0 ± 0.8 vs. 19.2 ± 0.6 years, p = 0.023). BMI was balanced between sexes (20.5 ± 1.8 vs. 20.2 ± 1.7 kg/m², p = 0.418). Non-obese males exhibited significantly higher systolic blood pressure than non-obese females (119.7 ± 14.3 vs. 109.2 ± 16.1 mmHg, p = 0.0005, Cohen's d = 0.68).

**Table 2:** Demographic, Anthropometric, and Electrocardiographic Characteristics of Non-Obese Participants Data presented as mean ± SD. *p < 0.05. ^†^Remains statistically significant after Bonferroni correction (α = 0.00625 for eight comparisons). BMI: body mass index; BP: blood pressure; QTc: heart rate-corrected QT interval; QTvar: beat-to-beat temporal QT variability in Lead II

Variable	Non-obese Males (n = 46)	Non-obese Females (n = 66)	Mean Difference (95% CI)	p-value	Cohen's d
Age (years)	19.0 ± 0.8	19.2 ± 0.6	-0.2 (-0.5 to 0.0)	0.023*	0.29
BMI (kg/m²)	20.5 ± 1.8	20.2 ± 1.7	0.3 (-0.4 to 0.9)	0.418	0.17
Systolic BP (mmHg)	119.7 ± 14.3	109.2 ± 16.1	10.6 (4.8 to 16.3)	0.0005*†	0.68
Diastolic BP (mmHg)	74.4 ± 8.9	71.9 ± 10.1	2.5 (-1.1 to 6.1)	0.184	0.26
Resting QTc (ms)	245.5 ± 88.4	268.5 ± 85.2	-23.0 (-56.3 to 10.3)	0.168	0.26
Resting QTvar (ms)	242.7 ± 118.9	240.8 ± 74.7	1.9 (-34.5 to 38.3)	0.915	0.02
Recovery QTc (ms)	247.7 ± 95.9	274.8 ± 101.7	-27.0 (-65.0 to 11.0)	0.159	0.27
Recovery QTvar (ms)	249.7 ± 124.0	219.3 ± 65.0	30.4 (-5.4 to 66.1)	0.094	0.30

Sex differences in QT interval and temporal beat-to-beat variability

Across both obese and non-obese groups, females exhibited numerically longer mean QTc durations than males at rest and during recovery; however, these differences did not achieve statistical significance in any comparison (p > 0.05, Cohen's d = 0.18-0.30).

In contrast, single-lead temporal beat-to-beat QTvar demonstrated marked sex differences specifically among obese individuals. Obese females exhibited significantly higher resting QTvar than obese males (227.2 ± 68.5 vs. 174.6 ± 57.2 ms, mean difference: -52.6 ms, 95% CI: -81.6 to -23.6 ms, p = 0.0002, Cohen's d = 0.83). This finding remained statistically significant after strict Bonferroni correction. This sex divergence persisted into the post-exercise recovery phase (214.0 ± 25.4 vs. 184.6 ± 81.6 ms, p = 0.045, Cohen's d = 0.48), although it did not withstand multiplicity correction at recovery.

Among non-obese participants, no significant sex differences in QTvar were detected at rest (240.8 ± 74.7 vs. 242.7 ± 118.9 ms, p = 0.915) or during recovery (219.3 ± 65.0 vs. 249.7 ± 124.0 ms, p = 0.094).

Correlations between BMI and QT parameters at rest

Correlations between BMI and resting electrocardiographic parameters are detailed in Table 3.

**Table 3:** Pearson Correlations Between BMI and QT Parameters at Rest *p < 0.05 (nominal significance); no correlations at rest remained significant following Bonferroni correction (α = 0.0031 for 16 total comparisons). BMI: body mass index; CI: confidence interval; QTc: heart rate-corrected QT interval; QTvar: beat-to-beat temporal QT variability in Lead II; r: Pearson correlation coefficient

Group	BMI vs. QTc (r (95% CI))	p-value	BMI vs. QTvar (r (95% CI))	p-value
Obese males (n = 54)	+0.208 (-0.068 to 0.454)	0.131	+0.031 (-0.240 to 0.299)	0.823
Obese females (n = 34)	+0.402* (0.072 to 0.647)	0.018	-0.290 (-0.564 to 0.053)	0.096
Non-obese males (n = 46)	+0.128 (-0.168 to 0.403)	0.396	-0.366* (-0.593 to -0.087)	0.012
Non-obese females (n = 66)	+0.283* (0.042 to 0.492)	0.021	-0.146 (-0.384 to 0.108)	0.242

At rest, nominal positive correlations were observed between BMI and QTc in obese females (r = 0.402, p = 0.018) and non-obese females (r = 0.283, p = 0.021), while males showed no significant association. A nominal negative correlation was found between BMI and QTvar in non-obese males (r = -0.366, p = 0.012). However, following Bonferroni correction for 16 comparisons (α = 0.0031), none of these resting correlations met statistical significance, indicating they should be considered exploratory associations.

Post-exercise recovery correlations

Correlations during post-exercise recovery are presented in Table 4.

**Table 4:** Pearson Correlations Between BMI and QT Parameters During Recovery *p < 0.05 (nominal significance). ^†^Remains statistically significant following Bonferroni correction (α = 0.0031 for 16 total comparisons). BMI: body mass index; CI: confidence interval; QTc: heart rate-corrected QT interval; QTvar: beat-to-beat temporal QT variability in Lead II; r: Pearson correlation coefficient

Group	BMI vs. QTc (r (95% CI))	p-value	BMI vs. QTvar (r (95% CI))	p-value
Obese males (n = 54)	+0.370* (0.111 to 0.582)	0.005	-0.166 (-0.421 to 0.111)	0.230
Obese females (n = 34)	+0.572*^†^ (0.298 to 0.756)	0.0004	-0.066 (-0.395 to 0.276)	0.710
Non-obese males (n = 46)	-0.008 (-0.299 to 0.284)	0.957	-0.325* (-0.562 to -0.037)	0.027
Non-obese females (n = 66)	+0.199 (-0.046 to 0.421)	0.109	-0.032 (-0.273 to 0.213)	0.798

Correlations between BMI and ventricular repolarization parameters during post-exercise recovery are shown in Figure 1. Following submaximal exercise, the positive correlation between BMI and recovery QTc in obese females (r = 0.572, p = 0.0004) remained statistically significant after Bonferroni correction (α = 0.0031). The correlation between BMI and recovery QTc in obese males (r = 0.370, p = 0.005) was nominally significant but fell above the strict Bonferroni threshold, representing an exploratory finding. Non-obese males continued to show a nominal negative correlation between BMI and QTvar (r = -0.325, p = 0.027), which was similarly non-significant after multiplicity adjustment.

## Discussion

This cross-sectional study examined sex differences and adiposity relationships in QT interval dynamics and single-lead temporal beat-to-beat QTvar at rest and following submaximal exercise. Our primary findings are (1) obese females demonstrated significantly greater beat-to-beat temporal QTvar than obese males at rest and post-exercise; (2) sex differences in QTc duration did not achieve statistical significance in either cohort; and (3) following submaximal exercise, a robust positive correlation emerged between BMI and recovery QTc in obese females that survived multiple-comparisons correction, with a parallel trend in obese males.

Sex differences in temporal beat-to-beat QT variability among obese individuals

The primary finding in our cohort was the elevated single-lead temporal QTvar observed in obese females compared to obese males (227.2 vs. 174.6 ms, Cohen's d = 0.83). Importantly, this sex difference was confined to the obese group and was absent in non-obese individuals, suggesting an interaction between sex and adiposity.

Methodologically, measuring QTmax - QTmin across 10 consecutive cycles in Lead II reflects temporal, beat-to-beat repolarization fluctuations rather than regional spatial dispersion across multiple ECG leads [10]. Elevated temporal beat-to-beat QTvar has been linked to dynamic repolarization instability, fluctuating autonomic inputs, and impaired repolarization reserve [10]. The elevated QTvar observed in obese young females may reflect an enhanced susceptibility of female ventricular myocardium to metabolic or autonomic disturbances associated with adiposity [11,12].

Temporal QT variability in non-obese vs. obese cohorts

A counterintuitive observation in our dataset was that non-obese participants demonstrated numerically higher resting temporal QTvar than obese males (242.7 ms in non-obese males vs. 174.6 ms in obese males). When evaluated as temporal beat-to-beat variability rather than spatial dispersion, this pattern is consistent with autonomic cardiovascular physiology [10]. Healthy, lean young adults typically exhibit prominent parasympathetic (vagal) tone and respiratory sinus arrhythmia at rest, producing marked cycle-to-cycle R-R interval changes that drive dynamic beat-to-beat QT adaptations across consecutive complexes [11,19,20]. In contrast, obesity is frequently characterized by blunted vagal modulation and resting sympathetic predominance, which reduces beat-to-beat heart rate variability and can constrain cycle-to-cycle QT fluctuations [4]. This autonomic rigidity likely explains the lower baseline temporal variability observed in obese males. Conversely, the elevated QTvar maintained by obese females (227.2 ms) may suggest an interaction where repolarization instability persists despite autonomic dampening, a hypothesis that requires dedicated autonomic testing.

Sex-specific QTc interval patterns

Although mean QTc intervals were numerically longer in females across all subgroups and conditions (by approximately 6-10%), these differences did not achieve statistical significance (p > 0.05). While the broader literature documents that adult females typically exhibit longer baseline QTc intervals due to androgen-mediated shortening of ventricular repolarization in males [11,12], our findings represent a statistically null result. We cannot exclude the possibility that subtle sex differences were present but undetected due to sample size constraints, particularly within the obese female cohort (n = 34). Consequently, these numerical variations should not be interpreted as evidence of a biological difference in this cohort, and larger investigations are required to establish whether subtle QTc differences interact with elevated beat-to-beat variability.

Correlations between BMI and repolarization parameters

Correlation analyses revealed differential associations between BMI and repolarization parameters across subgroups. At rest, positive correlations between BMI and QTc in females were nominally significant (r = 0.402 in obese, r = 0.283 in non-obese) but did not survive Bonferroni multiplicity adjustment (α = 0.0031).

Following submaximal exercise, the positive correlation between BMI and recovery QTc in obese females (r = 0.572, p = 0.0004) remained statistically robust after Bonferroni correction, while a corresponding positive correlation in obese males (r = 0.370, p = 0.005) represented an exploratory trend. In non-obese participants, exercise did not elicit positive BMI-QTc correlations. These findings suggest that acute physical exertion may unmask adiposity-associated repolarization prolongation during the post-exercise recovery window in individuals with elevated BMI.

Mechanistically, prior literature has suggested that systemic endocrine alterations, including altered levels of leptin, adiponectin, and sex steroid metabolism, could influence myocardial ion channel expression and repolarization dynamics [21,22]. However, because circulating hormone and adipokine concentrations were not measured in the present investigation, these proposed pathways remain speculative and strictly hypothesis-generating.

Clinical and practical considerations

The observed associations should be interpreted with a clinical perspective. The absolute mean QTc values observed across all subgroups remained below established pathological thresholds for long QT syndrome (>460 ms for females, >450 ms for males) [23]. Current clinical guidelines do not recommend routine resting or exercise ECG screening for asymptomatic young adults in the absence of clinical indications, syncope, or a family history of sudden death [24]. Our findings do not warrant changes to screening recommendations. Instead, they provide exploratory physiological data indicating that young adults with elevated adiposity - particularly females - exhibit differential repolarization recovery dynamics following submaximal exercise. These observations may help generate hypotheses for future prospective studies evaluating exercise safety and cardiovascular adaptations in obesity.

Limitations

This study has several important limitations that must be considered when interpreting the findings. 

Construct Validity of Electrocardiographic Parameter

Temporal beat-to-beat QTvar was derived from 10 consecutive cycles in Lead II (QTmax - QTmin) rather than spatial dispersion across a 12-lead ECG. As such, this parameter reflects dynamic cycle-to-cycle variability influenced by autonomic oscillations rather than regional spatial repolarization heterogeneity [25]. Furthermore, the reported values (174-250 ms) are substantially higher than typical spatial QT dispersion values (20-50 ms) because single-lead temporal ranges capture cycle-to-cycle rate adaptations over time. Direct comparisons with multilead spatial dispersion studies should not be made.

Subgroup Sample Size Imbalance

Although the overall sample size (N = 200) was adequate, recruitment through voluntary student enrollment resulted in an unbalanced subgroup distribution. Specifically, the obese female group (n = 34) fell below the calculated a priori minimum sample size of 39 participants per group. While post-hoc power remained moderate (≈ 74% for an effect size of d ≈ 0.57), this group was comparatively smaller, and findings within this subgroup warrant confirmation in larger, balanced cohorts.

Correction Formula and Calibration Considerations

Bazett's formula was utilized to allow comparisons with prior young adult literature, but it is known to overcorrect at higher heart rates [18,19]. Although recordings were obtained during minutes 10 to 15 of recovery when heart rates had partially returned toward baseline, residual tachycardia may have influenced recovery QTc values. Future exercise studies should include cubic-root corrections such as Fridericia's formula. In addition, mean absolute QTc values in this cohort (245.5-299.5 ms) were lower than conventional clinical ranges (380-450 ms), reflecting automated software caliper detection calibrated to the steepest T-wave downward slope rather than late isoelectric return. While internal consistency was maintained across all blinded comparisons, these absolute numbers should not be directly equated with manual clinical readings.

Absence of Mechanistic Biomarkers

Endocrine and metabolic biomarkers - including circulating leptin, adiponectin, free fatty acids, estradiol, and testosterone-were not measured. Consequently, mechanistic hypotheses regarding hormonal or adipokine modulation of ion channels could not be tested directly. 

Study Design

The cross-sectional design precludes drawing causal inferences regarding whether adiposity directly alters repolarization dynamics or whether shared constitutional factors influence both variables. 

Blinding and Physiological Cues

While participant identifiers and trial conditions were scrubbed from digital tracings and tracings were evaluated in randomized order, physiological signs of post-exercise recovery (such as mild residual tachycardia) may have provided contextual cues to observers during manual caliper adjudication.

## Conclusions

This cross-sectional study demonstrates significant sex differences in single-lead temporal beat-to-beat QTvar among young adults with obesity. Obese females exhibited significantly greater resting and post-exercise temporal QTvar compared to obese males, a difference not observed in non-obese cohorts. In addition, a statistically robust positive correlation between BMI and recovery QTc emerged following submaximal exercise in obese females. These findings highlight sex-specific electrophysiological patterns in young adults with obesity and emphasize the need for prospective, longitudinal studies using multilead spatial and temporal repolarization techniques to clarify their long-term clinical relevance.
